# A Preliminary Measurement and Understanding of Movement-Evoked Pain in African American Elder Adults

**DOI:** 10.1093/geroni/igaa057.2840

**Published:** 2020-12-16

**Authors:** Staja Booker

**Affiliations:** The University of Florida, Gainesville, Florida, United States

## Abstract

Osteoarthritis (OA) is a principal cause of disabling knee pain, and movement is a known exacerbator of pain in African Americans (AAs). Still, research has neglected to understand the relationship between pain with movement and its impact on function and mobility. Our previous study found significantly higher movement-evoked pain between AAs and White American (WAs). Therefore, this case-control observational study investigated inter-racial and intra-racial differences in movement-evoked pain in AAs and WAs (N= 28) who were 55-78 years-of-age (M= 65.75, SD= 6.23). We measured pain intensity (0-10) pre/ante/post multiple performance-based functional activities; we report preliminary results for 7-meter GAITRite® walk and Stair climbs. Pain intensity was higher before and after the 7-meter walk and stair climbs in AAs, although not significantly different than WAs. We will conduct additional statistical tests for the remaining functional activities to identify potential differences and ethnic-specific factors that distinguish movement-evoked pain and function by race.

